# Validation Study Methods for Estimating Odds Ratio in 2 × 2 × *J* Tables When Exposure is Misclassified

**DOI:** 10.1155/2013/170120

**Published:** 2013-12-23

**Authors:** Bijan Nouri, Najaf Zare, Seyyed Mohammad Taghi Ayatollahi

**Affiliations:** ^1^Department of Biostatistics, Faculty of Medicine, Shiraz University of Medical Sciences, Shiraz 7134845794, Iran; ^2^Department of Biostatistics, Infertility Research Center, Shiraz University of Medical Sciences, Shiraz 7134845794, Iran

## Abstract

*Background*. Misclassification of exposure variables in epidemiologic studies may lead to biased estimation of parameters and loss of power in statistical inferences. In this paper, the inverse matrix method, as an efficient method of the correction of odds ratio for the misclassification of a binary exposure, was generalized to nondifferential misclassification and 2 × 2 × *J* tables. *Methods*. Simple estimates for predictive values when misclassification is nondifferential are presented. Using them, we estimated the corrected log odds ratio and its variance for 2 × 2 × *J* tables, using the inverse matrix method. A two-step weighted likelihood method was also developed. Moreover, we compared the matrix and inverse matrix methods to the maximum likelihood (MLE) method using a simulation study. *Results*. In all situations, the inverse matrix method proved to be more efficient than the matrix method. Matrix and inverse matrix methods for nondifferential situations are more efficient than differential misclassification. *Conclusions*. Although MLE is optimal among all of the methods, it is computationally difficult and requires programming. On the other hand, the inverse matrix method with a simple closed-form presents acceptable efficiency.

## 1. Introduction

In epidemiology studies, where the assessment of the relationship between exposure and outcome variables is the main goal, misclassification of exposure variable leads to biased estimate of odds ratio. In multicenter clinical trials with an increasing number of centers, the possibility of misclassification of the exposure variable and the biases induced by it will arise. Methods to correct a possibly misclassified exposure so that the strength of the association between exposure and outcome variables can be precisely assessed have been a focus of statistical and epidemiological research for over 30 years. Beginning with classic papers, the issue of misclassification on tabular data has long been recognized and their adjustment has been considered [1–5]. In 1977, the matrix method was presented by Barron in order to correct nondifferential misclassification in 2 × 2 tables [6]. Greenland and Kleinbaum generalized it to differential misclassification and match paired data [7]. In addition, Greenland proposed a variance estimate for the matrix method under the assumptions of differential and nondifferential misclassifications [8]. Selen, in a simulation study, showed that both the matrix and the maximum likelihood methods performed equally well [9]. In 1990, Marshall presented a more direct inverse matrix approach for correcting differential misclassifications [10]. Morrissey and Spiegelman compared the matrix and inverse matrix methods to the maximum-likelihood estimator using a grid search for 2 × 2 tables [11]. He found that, under the assumption of differential misclassification, the inverse matrix method was always more efficient than the matrix method. Examples of validation studies in the misclassification context in linear models are prevalent in the statistical and epidemiologic literature [12–15]. More recent works are included in the reference section [16–20].

The main impediment to the use of the inverse matrix method, despite its superiority compared to the matrix method, is that it is restricted to differential misclassification and 2 × 2 tables, while, in practice, it is very likely that misclassification is nondifferential or that we have a 2 × 2 × *J* table, in which *J* determines the number of centers in a multicenter clinical trial or is the levels of a confounder [21]. For example, when misclassification is due to recall bias, it is expected that the rates of misclassification in case and control groups are the same, leading to nondifferential misclassification. Here, we aim to further extend the focus within the inverse matrix method when misclassification is nondifferential and data is stratified on *J* strata.

Section 2.1 provides definition and notation for a 2 × 2 × *J* table with a binary error-prone exposure. For the first time, we propose estimates for negative and positive predictive values as misclassification parameters under the assumption of nondifferential misclassification, which can be used to generalize an inverse matrix method to nondifferential cases (Section 2.2). Then we will generalize an inverse matrix approach to a nondifferential assumption and situations where the data is stratified on *J* strata (Section 3.1). Intuitive closed-form formulae for misclassification-adjusted effect and its variance are presented. The matrix method and the likelihood method will be briefly reviewed later. In addition, we will present a new two-step method in Section 3.4, which uses corrected cell count by the matrix method to construct a weighted maximum likelihood method. Finally, the study will continue with a simulation study that compares the mean square error (MSE) of each of the presented methods to the MLE under the assumptions of differential and nondifferential misclassifications. The main text focuses on simple formulae for conditions involving a binary misclassified exposure in a case-control study, with stratification on confounders. Several assumptions were made in this work. First, the disease status and level of strata were measured accurately. Second, the methods required an error-free criterion for exposure to validate the misclassified exposure in the validation study. Finally, we assumed that the main and validation studies are independent. The formulae are illustrated with data from an often-cited case-control study of sudden infant death syndrome (SIDS) and maternal use of antibiotics during pregnancy [22].

## 2. Method and Material

### 2.1. Definitions and Notations

To begin, consider a case-control study sample; binary exposure and outcome variables were measured by two error-prone and correct methods, respectively, as well as a *J* level variable *S* (*S* can be a confounder or a combination of some confounders). So, the data could be classified in a 2 × 2 × *J* table, for which its *j*th stratum contains *n*_1*j*_ cases and *n*_0*j*_ controls. Because the exposure status X*is an error-prone variable, the cross-table will be misclassified. The *j*th stratum of a 2 × 2 × *J* misclassified table and the notations that are employed in this paper are displayed in Table 1.

*X* is supposed to be an error-free variable that shows the actual status of the exposure. The sensitivity and specificity of the error-prone diagnosis of exposure are known as misclassification parameters or misclassification rates and defined by SE_*kj*_ = *P*(X* = 1 | *X* = 1, *Y* = *k*, *S* = *j*) and SP_*kj*_ = *P*(X* = 0 | *X* = 0, *Y* = *k*, *S* = *j*) for *k* = 0, 1 and *j* = 1, …, *J*, respectively. Also one can offer misclassification rates by another set of parameters, known as positive and negative predictive values, which can be defined as PPV_*kj*_ = *P*(*X* = 1 | X* = 1, *Y* = *k*, *S* = *j*) and NPV_*kj*_ = *P*(*X* = 0 | X* = 0, *Y* = *k*, *S* = *j*) for *k* = 0, 1 and *j* = 1, …, *J*, respectively. Among different approaches developed to deal with the misclassification problem, the inverse matrix method (direct method) uses positive and negative predictive values as misclassification parameters; on the other hand, the matrix method (indirect method) and the likelihood method both apply sensitivity and specificity values instead. Let *e*_kj_* = *P*(X* = 1 | *Y* = *k*, *S* = *j*) = *A*_kj_*/*n*_*kj*_ represents the prevalence of error-prone exposure for *k* = 0, 1 and *j* = 1, …, *J*, and also *f*_kj_* = 1 − *e*_kj_*.

Both of the sets of misclassifications are unknown, and it is required to estimate them through a validation study. Thus, in addition to the sample described above known as the main study, another random sample of size *m* is drawn separately. This sample makes up the external validation study. In order to estimate the misclassification parameters, as well as the error-prone status of exposure X*, the correct exposure status should be measured precisely for each subject of the validation sample by a dichotomous error-free variable *X*.

### 2.2. Estimation of Misclassification Parameters

Misclassification parameters are allowed to vary by levels of *J* in this approach and separate misclassification parameters for each stratum can be estimated, but if we notice that the misclassification rates are similar in strata, regardless of stratification, validation samples of strata should be combined. Due to the simplicity of notation, we will describe the case in which misclassification rates are the same across strata; otherwise, estimation of the parameters is similar.  Table 2 displays the available data *m*_*kli*_ for the validation study, where subscripts *k* = 0, 1, *l* = 0, 1, and *i* = 0, 1 indicate the outcome, error-free, and error-prone exposure status, respectively. Misclassification parameters and their variances are estimated in the validation study by the following formulae:

(1) $$\begin{array}{c} {\text{SE}}_{k}=\frac{m_{k11}}{m_{k1.}}\;{\text{SP}}_{k}=\frac{m_{k00}}{m_{k0.}},\\ \mathrm{Var}\left({\text{SE}}_{k}\right)=\frac{{\text{SE}}_{k}\left(1-{\text{SE}}_{k}\right)}{m_{k1.}},\\ \mathrm{Var}\left({\text{SP}}_{k}\right)=\frac{{\text{SP}}_{k}\left(1-{\text{SP}}_{k}\right)}{m_{k0.}},\\ {\text{PPV}}_{k}=\frac{m_{k11}}{m_{k.1}},\;\;{\text{NPV}}_{k}=\frac{m_{k00}}{m_{k.0}},\\ \mathrm{Var}\left({\text{NPV}}_{k}\right)=\frac{{\text{NPV}}_{k}\left(1-{\text{NPV}}_{k}\right)}{m_{k.0}},\\ \mathrm{Var}\left({\text{NPV}}_{k}\right)=\frac{{\text{NPV}}_{k}\left(1-{\text{NPV}}_{k}\right)}{m_{k.0}}. \end{array}$$

In this case, a point, “.”, in the subscripts stands for summation over that subscript. Misclassification is nondifferential when the misclassification rates are independent of the outcome status. In other words, when *P*(X* = 1 | *X* = 1, *Y* = *k*) = *P*(X* = 1 | *X* = 1) and *P*(X* = 0 | *X* = 0, *Y* = *k*) = *P*(X* = 0 | *X* = 0); otherwise, misclassification is differential. We can easily adapt estimates of sensitivity and specificity to the following nondifferential assumption:

(2) $$\begin{array}{c} \text{SE}=\frac{m_{.11}}{m_{.1.}},\;\;\text{SP}=\frac{m_{.00}}{m_{.0.}},\\ \mathrm{Var}\left(\text{SE}\right)=\frac{\text{SE}\left(1-\text{SE}\right)}{m_{.1.}},\\ \mathrm{Var}\left(\text{SP}\right)=\frac{\text{SP}\left(1-\text{SP}\right)}{m_{.0.}}. \end{array}$$

But once we assume that misclassification is nondifferential, the sensitivity and specificity are equal given the disease status. In this case, nondifferential sensitivity and specificity do not mean that PPV and NPV are equal across strata of the outcome because PPV and NPV are functions of exposure prevalence and sensitivity and specificity. Due to this problem, the methods that apply predictive values as misclassification parameters (such as the inverse matrix method) will be restricted to the differential assumption. In order to overcome this difficulty, Morrissey and spiegelman recommended the use of the same predictive values as those estimated by the differential misclassification assumption, which is not a reasonable approach [11]. Instead, in the case of nondifferential misclassification, we present the following estimates for predictive values:

(3) $$\begin{array}{c} {\text{PPV}}_{k}=\frac{\left(\text{SE}m_{k1.}\right)}{m_{k}^{\prime }}{,\;\;\text{NPV}}_{k}=\frac{\left(\text{SP}m_{k0.}\right)}{\left(m_{k..}-m_{k}^{\prime }\right)},\\ \mathrm{Var}\left({\text{PPV}}_{k}\right)=\frac{{\text{PPV}}_{k}\left(1-{\text{PPV}}_{k}\right)}{m_{k}^{\prime }},\\ \mathrm{Var}\left({\text{PPV}}_{k}\right)=\frac{{\text{NPV}}_{k}\left(1-{\text{NPV}}_{k}\right)}{\left(m_{k..}-m_{k}^{\prime }\right)}, \end{array}$$

where *m*_*k*_′ = (SE*m*_*k*1._ + (1 − SP)*m*_*k*0._). If sensitivity and specificity are constant and known, using the probability roles, we can estimate predictive values as follows:

(4) $$\begin{array}{c} {\text{PPV}}_{k}=\frac{{\text{SE}}_{k}\left({SP}_{k}-f_{k}^{*}\right)}{e_{k}^{*}D_{k}},{\;\;\text{NPV}}_{k}=\frac{{\text{SP}}_{k}\left({\text{SE}}_{k}-e_{k}^{*}\right)}{f_{k}^{*}D_{k}}. \end{array}$$

When equality of the misclassification parameters in *J* strata cannot be assumed, using distinct validation sample for each stratum and the above relations, misclassification parameters can be estimated separately.

**Example 1**

Table 3 exhibits the main study data from an often-cited case-control study on sudden infant death syndrome (SIDS), which has examined the relationship between maternal use of antibiotics during pregnancy and the odds of SIDS [22]. The table is classified on two strata (*J* = 2) according to the sex of infants, a common risk factor for SIDS. Drug use error-prone measurement was an interview response X*, and it was validated by medical records *X*. A separate sample from a validation study with dual exposure measurement (both error-prone and error-free) is presented in Table 2. Using (1)–(3) and the validation data, separate estimates with regard to both differential and nondifferential misclassification assumptions for sensitivity, specificity, and predictive values are presented in Table 4. Two cells in the last column of Table 4 are blank because, for nondifferential misclassification, sensitivity and specificity are the same for case and control groups.

## 3. Estimate of the Actual Association 

### 3.1. Inverse Matrix Method

As mentioned in the introduction, the inverse matrix method was proposed by Marshall to correct misclassified 2 × 2 tables [10]. He restricted the use of his method to the assumption of differential misclassification. According to the estimates we present for predictive values in (3), the inverse matrix method can be developed to the differential assumption. Now, we generalize this approach to 2 × 2 × *J* tables with misclassified exposure and either differential or nondifferential assumptions. Estimates for correct proportions of exposed and unexposed subjects in the *j*th stratum of the main study are *e*_*kj*_ = PPV_*kj*_ *e*_kj_* + (1 − NPV_*kj*_)*f*_kj_* and *f*_*kj*_ = NPV_*kj*_ *f*_kj_* + (1 − PPV_*kj*_)*f*_kj_*, respectively. These equations can be written in the matrix form as

(5) [e1jf1je0jf0j] =[PPV1j1−NPV1j001−PPV1jNPV1j0000PPV0j1−NPV0j001−PPV0jNPV0j]  ×[e1j∗f1j∗e0j∗f0j∗].

The biased log odds ratio estimate of the *j*th stratum is *θ*_j_* = log⁡(*A*_1j_*B_0j_*/*A*_0j_*B_1j_*), with asymptotic variance estimate *V*_j_* = ∑_*k*_(1/*A*_kj_* + 1/*B*_kj_*) where *k* = 0, 1. Corrected values in (5) are used to build the inverse matrix corrected log-odds-ratio *θ*_*j*_ = log⁡(*e*_1*j*_ *f*_0*j*_/*e*_0*j*_ *f*_1*j*_) where *e*_*kj*_ = *P*(*X* = 1 | *Y* = *k*, *S* = *j*) representing the estimate of actual prevalence of exposure for *k* = 0, 1 and *j* = 1, …, *J*, and also *f*_*kj*_ = 1 − *e*_*kj*_. Considering binomial distribution for *A*_kj_*, we derived the asymptotic variance for *θ*_*j*_ using the delta method as

(6) Vjj=Var⁡(θj)=∑k=0,1(dkj2Vekj∗+ekj∗2VPPVkj+fkj∗2VNPVkj)(fkj2ekj2),

where the letter *V* stands for an abbreviation for variance and *d*_*kj*_ = PPV_*kj*_ + NPV_*kj*_ − 1. If nondifferential misclassification is assumed, instead of the estimated positive and negative predictive values and their variance estimate in (1), corresponding values in (3) must be considered substitutes in expression (6) and also the corrected proportions in the disease group are no longer independent of the corrected proportions in the control group. Consequently, twice the covariance between log odds (7) must be subtracted from expression (6). Consider

(7) $$\begin{array}{c} \frac{V_{{\text{SE}}_{j}}}{f_{0j}f_{1j}D_{j}^{2}}+\frac{V_{{\text{SP}}_{j}}}{e_{0j}e_{1j}D_{j}^{2}}. \end{array}$$

If in the validation study for each stratum a separate misclassification-rate is estimated, the log odds ratios are independent; otherwise, using the same misclassification parameters for all the strata leads to covariance (8) between *θ*_*j*_ and *θ*_*i*_. Consider

(8) $$\begin{array}{c} V_{ij}=\sum_{k=0,1}^{}\frac{\left(V_{{\text{SE}}_{k}}/f_{kj}f_{ki}+V_{{\text{SP}}_{k}}/e_{kj}e_{ki}\right)}{D_{k}^{2}}. \end{array}$$

If the same misclassification parameters for all the strata are estimated by assuming nondifferential misclassification, the covariance estimate for *θ*_*j*_ and *θ*_*i*_ is given by

(9) $$\begin{array}{c} V_{ij}=\sum_{k=0,1\;}^{}\sum_{h=0,1}^{}{\left(-1\right)}^{h+k}\frac{\left(V_{\text{SE}}/f_{kj}f_{hi}+V_{\text{SP}}/e_{kj}e_{hi}\right)}{D^{2}}. \end{array}$$

Let *V* be the symmetric *J* by *J* variance-covariance matrix of the *J* estimated odds-ratio, with *ij*th element *V*_*ij*_. Then *θ*_*w*_ = (***θ***′*V* ^−1^1)*V*_*w*_ is the minimum variance weighted average of the estimated log-odds ratios, where *V*_*w*_ = 1/(1′*V* ^−1^1), and also 1 and ***θ*** are *J*-vector of ones and estimated odds ratios, respectively. For large strata sample, *θ*_*w*_ is a consistent uniformly asymptotic normal estimator for the actual log odds ratio, with consistent variance estimator *V*_*w*_. Hence, construction of a corrected Wald test of no association will be possible using the *z* statistic *θ*_*w*_/√*V*_*w*_, with a corresponding corrected 1 − *α* percent confidence interval for the actual log odds ratio as *θ*_*w*_ ± *z*_*t*=1−*α*/2_√*V*_*w*_, where *z*_*t*_ is the *t* = 1 − *α*/2 percentile of a standard normal distribution.

In order to evaluate the homogeneity of *θ*_*j*_, we can utilize an approximate *J* − 1 degree of freedom Chi-square statistics *X* ^2^ = ***θ***′*V* ^−1^ ***θ*** − *V*_*w*_^−1^ *θ*_*w*_^2^. Large values of this statistic indicate loss of homogeneity and so *θ*_*w*_ will be an inadequate summary of the association.

### 3.2. Matrix Method

Now we briefly outline the matrix method. In this method sensitivity and specificity are used as misclassification parameters to estimate the log-odds ratio. The matrix method uses the equations *A*_kj_* = SE_*kj*_ *A*_*kj*_ + (1 − SP_*kj*_)*B*_*kj*_ and *B*_kj_* = (1−SE_*kj*_)*A*_*kj*_ + SP_*kj*_ *B*_*kj*_ to estimate correct cell counts in the *j*th stratum; in matrix form these equations can be written as

(10) [A1jB1jA0jB0j]=[SE1j1−SP1j0                            01−SE1jSP1j0                            00000SE0j1−SE0j1−SP0jSP0j]−1 ×[A1j∗B1j∗A0j∗B0j∗].

The matrix corrected log-odds-ratio is *θ*_*j*_ = log⁡(*e*_1*j*_ *f*_0*j*_/*e*_0*j*_ *f*_1*j*_), *e*_*kj*_ = *A*_*kj*_/*n*_*kj*_ and *f*_*kj*_ = 1 − *e*_*kj*_. Considering binomial distribution for *A*_kj_*, Greenland provided the asymptotic variance for *θ*_*j*_, given by

(11) Vjj=Var⁡(θj)=∑k=0,1(VSEkj/fkj2+VSPkj/ekj2+ekj∗fkj∗/nkjekj2fkj2)(Dkj2),

where *D*_*kj*_ = SE_*kj*_ + SP_*kj*_ − 1. The other components of the variance-covariance matrix are similar to what we derived for the inverse matrix method.

This method has two limitations: when the sum of sensitivity and specificity is equal to one, the corrected count estimates are undefined because the misclassification matrix is singular in (10); if the sum is less than one, negative estimates for corrected cell counts occur.

#### 3.2.1. Direct Likelihood Method

Some authors use the maximum likelihood estimate (MLE) to estimate the actual odds ratio and test the exposure-disease association. Although this method can be the most efficient method of correction, it does not have a close form and requires an iterative solution to a nonlinear set of equations under some constraints. If the error-free exposure variable *X* was available, we would be able to estimate the covariate-adjusted odds ratio by the following multiple logistic regression model:

(12) $$\begin{array}{c} \text{logit}\left[\text{Pr}\left(Y=1\;|\;X,C\right)\right]=\alpha +\theta x+\sum_{j=1}^{J-1}\gamma_{j}c_{j}. \end{array}$$

However, we just have access to the error-prone binary variable X*. We assume that the covariates *C*_*j*_(*j* = 1,…, *J* − 1) are dummy variables that determine to which stratum a subject belongs. If the subject belongs to the *j*th stratum *C*_*j*_ = 1, as mentioned before, *θ* indicates overall log odds ratio. To obtain the ML estimate for unknown parameter *θ*, we should numerically maximize the following log likelihood function with respect to *θ*. The approximate standard error for this estimate is available by inversing the observed information matrix. Consider

(13) $$\begin{array}{c} l\left(\theta \right)=\sum_{j}^{}\sum_{k}^{}\sum_{i}^{}n_{ikj}\ln\left(\text{Pr}\left(X^{*}=i,Y=k\;|\;C_{j}=c\right)\right), \end{array}$$

where *n*_*ikj*_was presented in Table 1; note that Pr(X*, *Y* | **C** = *c*) in expression (13) is the observed data likelihood contribution in the likelihood function which can be obtained as follows:

(14) ∑x=01Pr(X∗=i ∣ X=l,Y=k,C=c)   ×Pr(Y=k ∣ X=l,C=c)Pr(X=l ∣ C=c),

where the first term in (14) can be obtained using the estimated sensitivity and specificity in (1) or (2) according to whether misclassification is differential or nondifferential, the second term displays the logistic model (12), and finally the last term is a nuisance parameter that can be modeled via a second logistic regression model of *X* on **C**.

### 3.3. A Weighted Likelihood Method

Now, we briefly present a two-step method which is a combination of the inverse matrix method and the likelihood method. In the first step, we use the inverse matrix method to correct misclassified tables, and in the second step, we utilize corrected cell count in the first step that is assigned to (*x*, *y*, *j*) log-likelihood contribution as weights. Formally, the weighted log-likelihood is given by

(15) $$\begin{array}{c} l\left(\theta \right)=\sum_{j=1\;}^{J}\sum_{k=0\;}^{1}\sum_{l=0}^{1}N_{lkj}l_{lkj}\left(\theta \right), \end{array}$$

where the *N*_*xyk*_ represents the corrected data cell count by the inverse matrix method and subscripts *l* = 0, 1, *k* = 0, 1, and *j* = 1, …, *J* indicate error-free exposure, outcome, and strata number, respectively. *N*_*lkj*_ represents the corrected data cell count by the inverse matrix method and *l*_*lkj*_(*θ*) is its log-likelihood contribution. This weighted method is simpler than the previous likelihood method and directly uses (*x*, *y*, *j*) log-likelihood contribution instead of (x*, *y*, *j*). The variance of corrected log-OR can be obtained by inversing the Fisher information matrix.

### 3.4. Combining the Results

Now, we need an approach to combine the results from the main study and the internal validation study. We emphasize that, for subjects in the validation study, a conventional analysis using an error-free exposure can be done to obtain a log-OR estimate *θ*_*v*_ with variance estimate *V*_*v*_. The estimate of the effect for the main study is biased and must be adjusted using the earlier formulae in order to obtain a corrected log-OR estimate *θ*_*m*_ with variance estimate *V*_*m*_. A weighted method to combine the log-OR from the two samples can be constructed as follows:

(16) $$\begin{array}{c} \theta_{c}=\frac{\left(\theta_{m}V_{v}+\theta_{v}V_{m}\right)}{\left(V_{m}+V_{v}\right)}. \end{array}$$

*θ*_*c*_ has variance estimate *V*_*c*_ = *V*_*m*_ *V*_*v*_/(*V*_*m*_ + *V*_*v*_). The weighted method proceeds well if *θ*_*v*_ and *θ*_*m*_ estimate a common value, and this is achieved when samples are randomly selected from the population, and the misclassification parameter estimates are unbiased.

**Example 2**

For the data in Table 3, we have misclassified OR = 1.124 and ln⁡(OR) = 0.1174(0.02357), in which the term in the bracket represents the estimated variance. Suppose that the investigator assumes that misclassification rates are equal in both the strata of male and female infants. Under the nondifferential assumption and using the parameters estimated in Table 4, the matrix method yields $\hat{\text{OR}}=1.26$ and $\ln\left(\hat{\text{OR}}\right)=0.2311\left(0.04305\right)$, and the inverse matrix method yields $\hat{\text{OR}}=1.237$ and $\ln\left(\hat{\text{OR}}\right)=0.213\left(0.04103\right)$. In contrast, we supposedly have differential estimates for misclassification rates in Table 2; thus, the results from the matrix and inverse matrix method will be $\ln\left(\hat{\text{OR}}\right)=0.2678\left(0.05147\right)$ and $\ln\left(\hat{\text{OR}}\right)=0.2471\left(0.04012\right)$, respectively.

Applying the direct ML (13) to the data in Table 3 yields $\hat{\beta }=\ln\left(\hat{\text{OR}}\right)=0.00585\left(0.02418\right)$ and $\hat{\beta }=\ln\left(\hat{\text{OR}}\right)=0.505\left(0.0312\right)$ under the differential and nondifferential assumptions, respectively. Note the dramatic shift in the implication of assuming a differential misclassification. The application of the two-step weighted likelihood approach produces $\hat{\beta }=\ln\left(\hat{\text{OR}}\right)=0.3027\left(0.048309\right)$ and $\hat{\beta }=\ln\left(\hat{\text{OR}}\right)=0.3047\left(0.04831\right)$ given the differential and nondifferential cases, respectively.

## 4. Simulation Studies

We conducted a simulation study to compare corrected log-odds-ratios obtained from both matrix and inverse matrix methods and also to assess the appropriateness of the maximum likelihood and weighted maximum likelihood for 2 × 2 × *J* tables. In order to mimic the SIDS example, a total of 1000 sets of data were generated with a binary outcome (*Y*) and also two other binary variables as error-free exposure status and stratification indicator (*X* and *S*) and a total sample size of 1144. Correct cell counts for each table were generated with regard to the following simulation conditions: prevalence of *P*(*S* = 1) = 0.5 and conditional prevalence of exposure are *P*(*X* = 1 | *S* = 1) = 0.5 and *P*(*X* = 1 | *S* = 0) = 0.5. In order to generate the response variable, the binary distribution with parameter *π*_*lj*_ = exp⁡⁡(*α* + *θx*_*l*_ + *γS*_*j*_)/(1 + exp⁡⁡(*α* + *θx*_*l*_ + *γS*_*j*_)) was used, where we set the effect parameters for all the simulation scenarios as *α* = −1, *θ* = 1.71 and *γ* = 1. The misclassified exposure (X*) was generated assuming numerous nondifferential conditions. In order to estimate the misclassification parameters for every one of the simulated data sets, three scenarios of 20%, 30%, and 40% of each of their strata were taken as internal validation samples separately. The programming code could be provided via request from the authors.

The last four columns of Table 5 represent different scenarios related to cases in which nondifferential misclassifications vary across strata, while the four columns before them display various situations in which nondifferential misclassifications are constant across strata; analysis of each column is in accordance with the manner in which that column was generated. For the purpose of comparing the different methods, the mean square error (MSE) measure was used in order to take into account the bias and variance of estimators simultaneously. As you can see, the MLE estimator is more efficient than other estimator methods in all scenarios. Our simulation study showed that for nondifferential misclassification the performance of these inverse matrix methods and likelihood method is very close but is not equivalent. The inverse matrix method is significantly more precise than the matrix method. Performance of the likelihood and weighted likelihood methods is relatively the same under both assumptions. The efficiency of all estimators would be improved by increasing the validation study sample size, but this improvement would be more prominent in the weighted likelihood method, as the efficiency of the estimator will be approximately doubled by increasing the validation study sample size.

## 5. Discussion and Conclusions

The misclassification of exposure is an issue of broad concern in epidemiological research studies, and considerable amount of pieces of literature discuss adjusting inferences on exposure-disease relationships regarding such misclassification. Matrix method, inverse matrix method, likelihood method, Bayesian method, and SIMEX method are some of the general tools for performing such adjustments. Our main focus was on the development of the inverse matrix method to nondifferential misclassification and 2 × 2 × *J* tables. At least in the context of misclassified binary exposure, this paper has illustrated several positive attributes of the inverse matrix method. First, to assess the diagnostic tests, the importance of predictive values (rather than sensitivity and specificity) is well known. The inverse matrix method, as a direct method, uses the predictive values. We presented simple closed-form estimates for positive and negative predictive values when misclassification is nondifferential. Second, the simulation study showed that the inverse matrix method estimator is practically more precise than the matrix method in all scenarios.

We also examined the effect of the parameters controlled by the researcher on the MSEs of these approaches. We found that the optimal estimator depends on the size of the validation study. This was due to the fact that, in some cases where the validation sample is large, a good part of the information about *θ* can come from the validation study. Morrissey and spiegelman noted this phenomenon previously from the comparison of these methods for 2 × 2 tables. These researchers showed that the efficiency of estimators depends more on the relative size of the validation study than on the case-control ratio [11]. In another study, Greenland noted that the samples from which the misclassification parameters are estimated must be large enough to assure the approximate normality of SE, SP, PPV, and NPV [8]. Lyles noted clearly that under the differential misclassification setting, the inverse matrix method is equivalent to the maximum likelihood approach [19]. There are closed-form solutions for both, which are exactly the same. For this reason, both approaches should be equally more efficient than the matrix method.

The methods described in this study handle the misclassification of exposure, but they are easily adapted for disease misclassification. The study depends on some assumptions stated at the end of the introduction. Further studies seem to be necessary to compare and extend these methods, as those assumptions are not met. This would make sense when the outcome status and exposure status are simultaneously misclassified and when there is not a gold standard to validate the exposure.

In conclusion, when trying to make a decision about which method to be used, it can be helpful to consider the following concept. Although the MLE method has minimum MSE in all the scenarios, it can be computationally difficult and does not have a simple closed-form expression like the matrix and inverse matrix methods. When misclassification is non-differential, the inverse matrix method performs very well. Even when the size of the validation study is large enough, it can perform equal to the likelihood method. The two-step likelihood method has a simpler form than the likelihood method and can perform for nondifferential misclassification like the matrix and inverse matrix methods.

## Figures and Tables

**Table 1:** Notation for *j*th stratum of a 2 × 2 × *J* misclassified table.

	Error-prone exposure (X*)		
Outcome (*y*)	1	0	
1	*A*_1j_* (*n*_11*j*_)	*B*_1j_* (*n*_01*j*_)	*n*_1*j*_
0	*A*_0j_* (*n*_10*j*_)	*B*_0j_* (*n*_00*j*_)	*n*_0*j*_

**Table 2:** Data layout for the validation study.

Validation study				
	*Y* = 1		*Y* = 0	
X*	*X* = 1	*X* = 0	*X* = 1	*X* = 0
1	*m*_111_ = 29	*m*_101_ = 22	*m*_011_ = 21	*m*_001_ = 12
0	*m*_110_ = 17	*m*_100_ = 143	*m*_010_ = 16	*m*_000_ = 168

*m*_*kli*_.

**Table 3:** Uncorrected data from SIDS study stratified on the sex of infant.

	Males		Females	
Interview response (X*)	*Y* = 1	*Y* = 0	*Y* = 1	*Y* = 0
Use	80	42	42	59
No use	257	261	185	218

Total	**337**	**303**	**227**	**277**

**Table 4:** Estimates of misclassification parameters and their variance.

	Differential	Nondifferential
PPV_1_	0.5686 (0.0048096)	0.6300 (0.0053002)
PPV_0_	0.6363 (0.0070127)	0.5567 (0.0061395)
NPV_1_	0.8937 (0.0005937)	0.8904 (0.0005842)
NPV_0_	0.9130 (0.0004316)	0.9168 (0.0004310)
SE_1_	0.6304 (0.0050651)	0.6024 (0.0028857)
SE_0_	0.5676 (0.0066332)	
SP_1_	0.8667 (0.0007003)	0.9014 (0.0025761)
SP_0_	0.9333 (0.0003457)	

These estimates are based on the assumption that misclassification rates are the same on male and female strata.

**Table 5:** Simulation results for a 2 × 2 × 2 table when binary exposure is misclassified.

Validation sample rate	Method	Strata 1	Se_11_ = 0.90 Sp_11_ = 0.90 Se_10_ = 0.90 Sp_10_ = 0.90	Se_11_ = 0.85 Sp_11_ = 0.90 Se_10_ = 0.85 Sp_10_ = 0.90	Se_11_ = 0.80 Sp_11_ = 0.85 Se_10_ = 0.80 Sp_10_ = 0.85	Se_11_ = 075 Sp_11_ = 080 Se_10_ = 075 Sp_10_ = 0.80	Se_11_ = 0.90 Sp_11_ = 0.90 Se_10_ = 0.90 Sp_10_ = 0.90	Se_11_ = 0.85 Sp_11_ = 0.90 Se_10_ = 0.85 Sp_10_ = 0.90	Se_11_ = 0.80 Sp_11_ = 0.85 Se_10_ = 0.80 Sp_10_ = 0.85	Se_11_ = 0.70 Sp_11_ = 0.80 Se_10_ = 0.70 Sp_10_ = 0.80
		Strata 2	Se_21_ = 0.90 Sp_21_ = 0.90 Se_20_ = 0.90 Sp_20_ = 0.90	Se_21_ = 0.85 Sp_21_ = 0.90 Se_20_ = 0.85 Sp_20_ = 0.90	Se_21_ = 0.80 Sp_21_ = 0.85 Se_20_ = 0.80 Sp_20_ = 0.85	Se_21_ = 075 Sp_21_ = 080 Se_20_ = 075 Sp_20_ = 0.80	Se_21_ = 0.85 Sp_21_ = 0.85 Se_20_ = 0.85 Sp_20_ = 0.85	Se_21_ = 0.80 Sp_21_ = 0.85 Se_20_ = 0.80 Sp_20_ = 0.85	Se_21_ = 0.70 Sp_21_ = 0.80 Se_20_ = 0.70 Sp_20_ = 0.80	Se_21_ = 0.90 Sp_21_ = 0.95 Se_20_ = 0.90 Sp_20_ = 0.95
*n*_*v*_ = 0.2	Matrix		1.717 (0.0298)	1.711 (0.0325)	1.671 (0.0400)	1.652 (0.0483)	1.720 (0.0301)	1.713 (0.0338)	1.682 (0.0418)	1.715 (0.0375)
	Inverse matrix		1.724 (0.0232)	1.723 (0.0234)	1.732 (0.0238)	1.686 (0.0240)	1.727 (0.0218)	1.731 (0.0232)	1.634 (0.0252)	1.762 (0.0233)
	Likelihood		1.723 (0.0146)	1.725 (0.0155)	1.730 (0.0176)	1.689 (0.0182)	1.724 (0.0165)	1.730 (0.0182)	1.682 (0.0203)	1.724 (0.0187)
	Weighted likelihood		1.737 (0.0560)	1.726 (0.0554)	1.730 (0.0561)	1.685 (0.0539)	1.738 (0.0534)	1.730 (0.0532)	1.691 (0.0560)	1. 721 (0.0544)

*n*_*v*_ = 0.3	Matrix		1.718 (0.0247)	1.720 (0.0263)	1.721 (0.0300)	1.659 (0.0358)	1.721 (0.0272)	1.711 (0.0330)	1.694 (0.0362)	1.718 (0.0335)
	Inverse matrix		1.720 (0.0188)	1.723 (0.0194)	1.723 (0.0237)	1.678 (0.0310)	1.723 (0.0223)	1.729 (0.0240)	1.673 (0.0322)	1.728 (0.0232)
	Likelihood		1.719 (0.0140)	1.719 (0.0144)	1.716 (0.0178)	1.671 ) 0.0174(	1.711 (0.0145)	1.722 (0.0181)	1.696 (0.0181)	1.723 (0.0173)
	Weighted likelihood		1.727 (0.0372)	1.740 (0.0383)	1.745 (0.0431)	1.682 ) 0.0364(	1.721 (0.0375)	1.728 (0.0388)	1.624 (0.0443)	1.733 (0.0362)

*n*_*v*_ = 0.4	Matrix		1.714 (0.0214)	1.719 (0.0228)	1.713 (0.0249)	1.662 (0.0293)	1.718 (0.0224)	1.712 (0.0235)	1.697 (0.0250)	1.711 (0.0233)
	Inverse matrix		1.712 (0.0162)	1.725 (0.0167)	1.723 (0.0140)	1.684 (0.0178)	1.715 (0.0166)	1.727 (0.0165)	1.692 (0.0162)	1.729 (0.0165)
	Likelihood		1.710 (0.0132)	1.722 (0.0140)	1.716 (0.0147)	1.673 (0.0163)	1.710 (0.0131)	1.716 (0.0140)	1.688 (0.0142)	1.711 (0.0162)
	Weighted likelihood		1.726 (0.0280)	1.724 (0.0278)	1.727 (0.0275)	1.678 (0.0277)	1.729 (0.0281)	1.728 (0.0282)	1.674 (0.0275)	1.729 (0.0273)

Numbers in each cell reflect mean of adjusted odds ratios (MSE) based on 1000 simulated data sets, with true OR = 1.71.
